# Understanding of the Viscoelastic Response of the Human Corneal Stroma Induced by Riboflavin/UV-A Cross-Linking at the Nano Level

**DOI:** 10.1371/journal.pone.0122868

**Published:** 2015-04-01

**Authors:** Cristina Labate, Maria Penelope De Santo, Giuseppe Lombardo, Marco Lombardo

**Affiliations:** 1 Department of Physics, University of Calabria, Ponte P. Bucci, Cubo 33B, 87036, Rende, Italy; 2 Consiglio Nazionale delle Ricerche, Istituto di Processi Chimico-Fisici, Unit of Support Cosenza, Ponte P. Bucci, Cubo 33B, 87036, Rende Italy; 3 Consiglio Nazionale delle Ricerche, Istituto di Processi Chimico-Fisici, Viale Stagno D’Alcontres 37, 98158, Messina, Italy; 4 Vision Engineering Italy srl, Via Adda 7, 00198 Rome, Italy; 5 Fondazione G.B. Bietti IRCCS, Via Livenza 3, 00198 Rome, Italy; Cardiff University, UNITED KINGDOM

## Abstract

**Purpose:**

To investigate the viscoelastic changes of the human cornea induced by riboflavin/UV-A cross-linking using Atomic Force Microscopy (AFM) at the nano level.

**Methods:**

Seven eye bank donor corneas were investigated, after gently removing the epithelium, using a commercial AFM in the force spectroscopy mode. Silicon cantilevers with tip radius of 10 nm and spring elastic constants between 26- and 86-N/m were used to probe the viscoelastic properties of the anterior stroma up to 3 µm indentation depth. Five specimens were tested before and after riboflavin/UV-A cross-linking; the other two specimens were chemically cross-linked using glutaraldehyde 2.5% solution and used as controls. The Young’s modulus (*E*) and the hysteresis (*H*) of the corneal stroma were quantified as a function of the application load and scan rate.

**Results:**

The Young’s modulus increased by a mean of 1.1-1.5 times after riboflavin/UV-A cross-linking (P<0.05). A higher increase of *E*, by a mean of 1.5-2.6 times, was found in chemically cross-linked specimens using glutaraldehyde 2.5% (P<0.05). The hysteresis decreased, by a mean of 0.9-1.5 times, in all specimens after riboflavin/UV-A cross-linking (P<0.05). A substantial decrease of *H*, ranging between 2.6 and 3.5 times with respect to baseline values, was observed in glutaraldehyde-treated corneas (P<0.05).

**Conclusions:**

The present study provides the first evidence that riboflavin/UV-A cross-linking induces changes of the viscoelastic properties of the cornea at the scale of stromal molecular interactions.

## Introduction

Corneal cross-linking via UV-A irradiation of the cornea permeated with riboflavin, that acts as a photosensitizer, is an established procedure aimed at slowing down or halting the progression of keratoconus [1,2]. Several imaging techniques have been applied for demonstrating the corneal structural alterations of keratoconus corneas [3,4]. The studies showed little or no lamellar interweaving in the anterior stroma, involving disinsertion of lamellae from Bowman’s layer. In the posterior stroma, they showed an uneven loss of orthogonal interlacing collagen fibers. The changes observed in these structures may be responsible in part for the changes in the mechanical properties of keratoconus corneas that lead directly to altered corneal shape.

Riboflavin/UVA corneal cross-linking works by improving the biomechanical properties of corneal tissue and by increasing its resistance to enzymatic digestion [5–7]. The exact molecular mechanisms of corneal cross-linking are still under investigation. They are dependent upon the presence of carbonyl groups and oxygen and may include the formation of additional intra-molecular collagen cross-linking bonds, additional bonds between the collagen molecules at fibril surface and additional bonds between collagen and proteoglycans core proteins [8–10]. In clinic, evidences of treatment efficacy are based on stabilization of corneal topography, pachymetry and refraction over time [11].

Various experimental methodologies, which included strip extensiometry, inflation testing of whole eye globes, Brilloun microscopy, scanning acoustic microscopy and atomic force microscopy (AFM) [12–17], have been used to investigate the biomechanical changes of the corneal tissue induced by riboflavin/UV-A cross-linking globally or locally.

Atomic force microscopy has been demonstrated to reliably characterize the biomechanics of the corneal tissue either at the micro- or nano-level, providing information about the local elastic or viscoelastic properties of the tissue [16–20]. Recently, the technique has been shown to investigate the stiffening effect induced by riboflavin/UV-A cross-linking on porcine and human corneal tissues [16,17].

The scope of our study was to provide a quantitative measurement of the viscoelastic changes induced by riboflavin/UV-A cross-linking on the anterior stroma using AFM with nanometer-sized tip. The study protocol allowed us to investigate the corneal stroma on areas that were comparable with the dimension of the collagen fiber (30 nm width).

## Materials and Methods

### Corneal tissues and cross-linking procedures

Seven human sclero-corneal tissues, from different donors, not suitable for transplantation, were obtained from the Veneto Eye Bank Foundation (Venezia Zelarino, Italy; www.fbov.org). Written informed consent from the next of kin was obtained for the use of samples in research. All human tissues were used in compliance with the guidelines of the Declaration of Helsinki for research involving the use of human tissue and the experimental protocol was approved by the National Research Council (CNR) research ethics and bioethics advisory committee. Donors did not have history of corneal pathologies or eye surgery. The sclero-corneal tissues were explanted between 10 and 19 hours after death and immediately preserved at 4° C in dextran-enriched storage medium, after gently removing the epithelium with a Merocel sponge (Medtronic, Minneapolis, MN, USA) soaked in deionised water. Corneal thickness and endothelial cell density were measured using an ultrasound contact pachymetry (UP-1000, Nidek Co. Ltd, Gamagori, Japan) and an inverted optical microscopy (Axiovert 25, Carl Zeiss Microscopy, Jena, Germany) respectively. The donor characteristics are summarized in Table 1.

**Table 1:** Characteristics of the eye bank donor corneal tissues.

	**Group 1: riboflavin/UV-A corneal cross-linking**					**Group 2: glutaraldehyde 2.5% cross-linking**	
	**Cornea 1**	**Cornea 2**	**Cornea 3**	**Cornea 4**	**Cornea 5**	**Cornea 6**	**Cornea 7**
**Donor age** (years)	69	70	69	69	53	68	63
**Endothelial Cell Density** (cells/mm^2^)	2700	1900	2600	2200	2300	1900	2100
**Central corneal thickness** (μm)	501	547	507	545	520	537	561

The corneal specimens were divided in two groups: the first group of five specimens underwent riboflavin/UV-A cross-linking treatment; the second group of two specimens underwent chemical cross-linking using glutaraldehyde 2.5% solution and was used as control. Indeed, chemical cross-linking offers a direct method of identifying stable protein interactions; it is the ideal strategy for an unambiguous demonstration of protein-protein interactions, *in vitro*.

The viscoelastic properties of each specimen were measured before and 12 hours after the cross-linking procedures. Before commencing the experiment, each specimen was trephined to 9.00 mm diameter using a Barron donor punch (Coronet, Network Medical Products Ltd, Ripon, UK) and then immersed in 20% dextran solution for 2 hours. The riboflavin/UV-A corneal cross-linking was performed as follows: each corneal lenticule was immersed in 20% dextran-enriched 0.1% riboflavin solution (Ricrolin, Sooft Italia SpA, Montegiorgio, Italy) for 30 minutes, under dim light conditions to avoid effects of incident light [21]. Before UV-A irradiation, the specimens were gently washed with balanced physiological solution (BPS) in order to remove the excess of riboflavin. Each corneal lenticule was then irradiated with UV-A (370±8 nm) for 30 minutes with an irradiance of 3 mW/cm^2^. The UV-A delivery system (Vega, CSO srl, Scandicci, Italia) was located 56 mm from the stromal surface. An irradiation area of 8.0 mm diameter was used in all cases. After UV-A irradiation, the corneal specimens were kept in 20% dextran solution overnight.

The second group of corneal specimens was immersed in glutaraldehyde 2.5% solution for 30 minutes. After gently washing the corneal lenticules with BPS, they were kept in 20% dextran solution overnight.

### Atomic force microscopy data acquisition

Before and 12 hours after cross-linking, each corneal tissue was gently placed on a specially designed Teflon environmental cell with the endothelial side facing downward and kept in place without the use of glue to preserve its mechanical properties [18]. These were measured using a NanoScope IIIa Atomic Force Microscope (Veeco-Bruker) in the Force Spectroscopy mode [12,18]. Measurements were performed at 27°C (close to the physiological temperature of the human cornea), with the specimens immersed in 20% dextran solution, using phosphorus doped rectangular silicon cantilevers of nominal elastic constant between 20- and 80-N/m (TESPA, Bruker). Dextran was used as imaging medium to keep the tissues at the same osmolarity before and after riboflavin/UV-A corneal cross-linking. The nominal value of the tip radius of curvature was 10 nm. Force curves were obtained on three different locations at the center of the anterior stroma of each sample and several force curves (30 curves for each rate) were recorded at four different approach speeds, i.e., 1.7 μm/s, 3.5 μm/s, 8.2 μm/s and 12.3 μm/s, at each location. No adhesion between the tip and the sample surface was detected in any sample. We made sure to avoid any possible alteration of the tissue during measurement; any rupture in the material would have induced a discontinuity in the force curve profile and therefore would have been clearly identified.

### Atomic force microscopy data analysis

The AFM data interpretation requires the correct calibration of both the AFM optical sensitivity and the spring constant of each cantilever [18]. Before experimental investigations, each cantilever was measured using a scanning electron microscope (FEI Quanta 400, OR, USA) to determine its physical constants, such as the length, width and thickness in order to evaluate its second moment such as:

$$I_{trapezoid}=\frac{t^{3}\left(4ab+b^{2}\right)}{36\left(a+b\right)}$$ (1)

where *t* is the cantilever’s thickness, *a* is the width of the top face of the lever, and *b* is the width of the bottom face. The optical detector sensitivity was then measured as the slope of the force curve in 20% dextran, when the tip was in contact with a rigid surface as mica; this value was used to convert the cantilever deflection, in Volts (V), to deflection in micrometers (μm) by using a purpose-written routine in Matlab (ver. 2013; The Mathworks, Inc., Natick, MA). The same conditions were maintained during each experimental run performed using the same cantilever.

The spring constant (*k*) was, then, calculated from [18]:

$$k=\frac{3E_{k}I_{trapezoid}}{L^{3}}$$ (2)

where *L* is the length of the reference cantilever, *E*_*k*_ is the spring’s elastic modulus and *I*_*trapezoid*_ is the moment of inertia of the cantilever. From these experimental calibrations, the spring constants of the cantilevers used in the experiments were in the range between 26 N/m and 86 N/m (42-, 86-, 26-, 47-, 67-, 48- and 56- N/m for samples n.1 to n.7 respectively).

**Fig 1:**
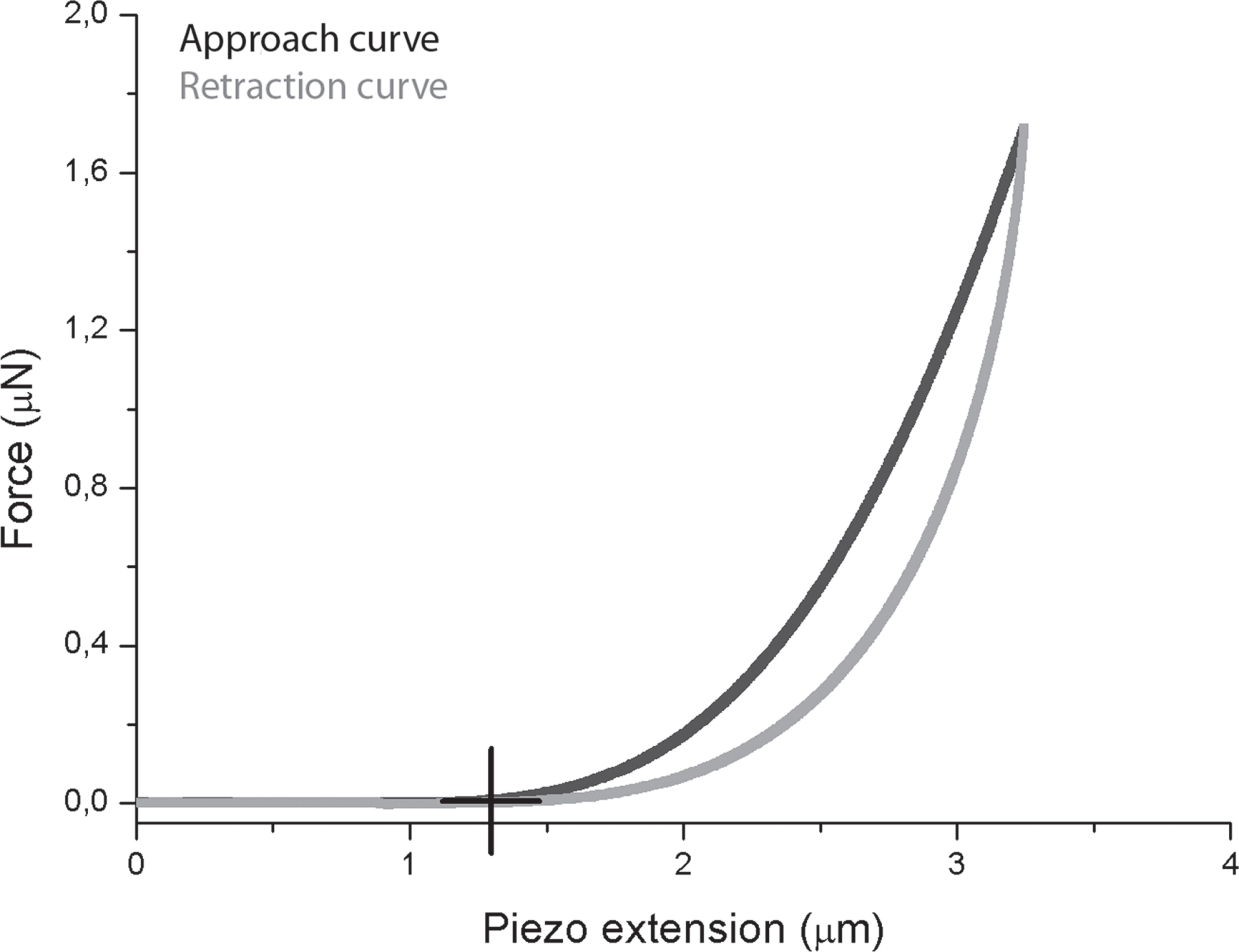
Sketch of a typical force-displacement curve using nanometer-sized tip. The black curve shows the force experienced by the nanometer-sized tip when approaching the sample, while the grey curve represents the force experienced on retracting from the sample. The main interaction force between the tip and the corneal specimen is the short-ranged van der Waals force. The black cross identifies the contact point; the absence of “jump-to-contact” indicates no adhesion between the tip and the stromal surface in 20% dextran solution. As the tip comes into contact with the stroma, there is a gradual increase in the deflection of the cantilever, as expected for soft biological specimens. In this study, the Young’s modulus was calculated by fitting the approach curve with the Hertz-Sneddon model for a conical indenter. Upon retraction, the approach and retraction curves do not overlap: this phenomenon is due to stromal hysteresis.

The Young’s modulus of elasticity (*E*) and the hysteresis (H) were calculated individually for each corneal sample before and after cross-linking. The Young’s modulus was calculated by fitting the approach curve with the Hertz-Sneddon model for a conical indenter [18,22,23]. This model relates the loading force to indentation, which for a conical indenter is:

$$F=F_{0}+\frac{2}{\pi }\frac{E}{1-\nu^{2}}{\left(\delta -\delta_{0}\right)}^{2}\tan\left(\alpha \right)$$ (3)

where *F* is the loading force in Newton (N), *v* is Poisson’s ratio (0.49 for the corneal tissue), δ is the indentation depth, *E* is the Young’s modulus in Pascal, F_0_ and δ_0_ are the loading force at baseline and the indentation depth at the contact point, respectively. The custom software permitted to identify automatically the contact point and thus to provide a reproducible value of *E*, as previously shown [18]. The hysteresis was calculated as the area encircled between the extending and retraction curves (between the point of maximum deflection and the contact point). A typical force curve acquired when probing the anterior stroma with a nanometer-sized tip is schematized in Fig 1.

### Statistics

A commercial software program (KyPlot, KyensLab Inc., Tokyo, Japan) was used for statistical testing. Data are given as mean ± standard deviation. Each value of *E* or *H* refers to an average on three different areas of each corneal lenticule. The Wilcoxon signed rank test for paired data was used to statistically compare the changes of *E* and *H* induced by cross-linking in each specimen. The sample size and statistical test were calculated (GPower, ver 3.1.2) having 81% power to determine differences of 0.23 MPa and 0.05 pJ between pre- and post-treatment *E* and *H* values respectively. The differences with a *P* value of 0.05 or less were considered statistically significant.

## Results

### Young’s modulus

**Fig 2:**
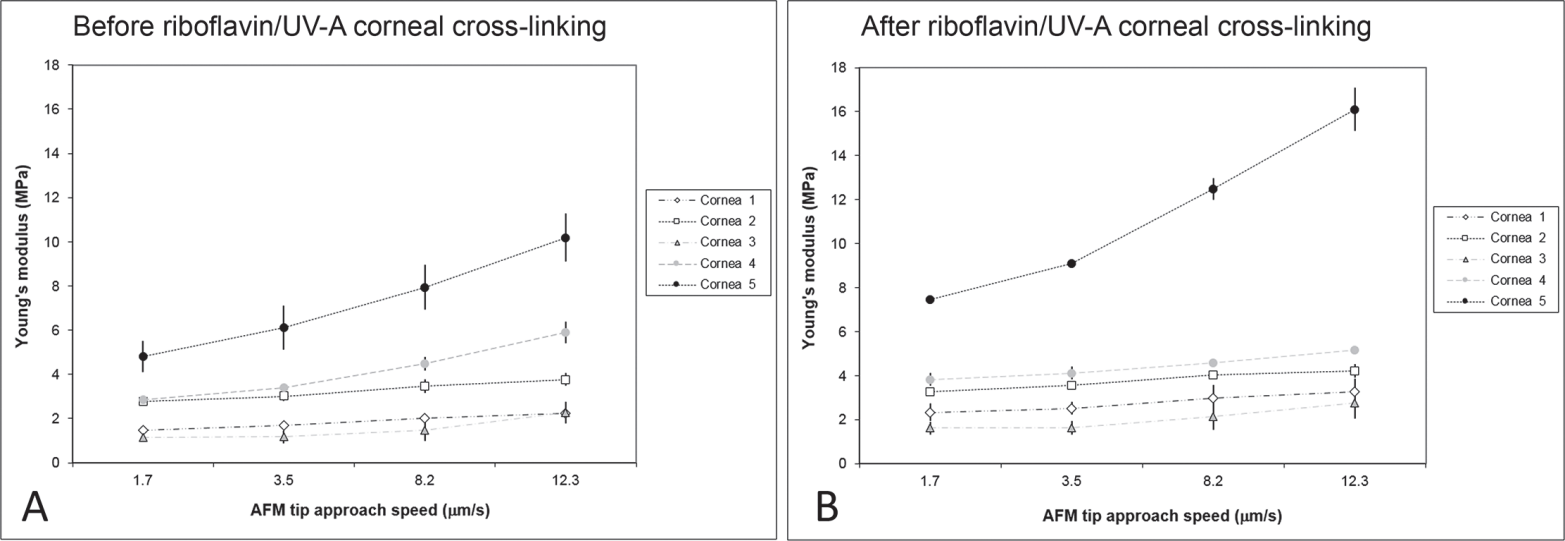
Plots of the Young’s modulus before and after riboflavin/UV-A corneal cross-linking. A) Average values of the Young’s modulus at different approach speeds in all five corneal specimens before riboflavin/UV-A corneal cross-linking. B) Average values of the Young’s modulus after treatment. Bars indicate standard deviation.

**Fig 3:**
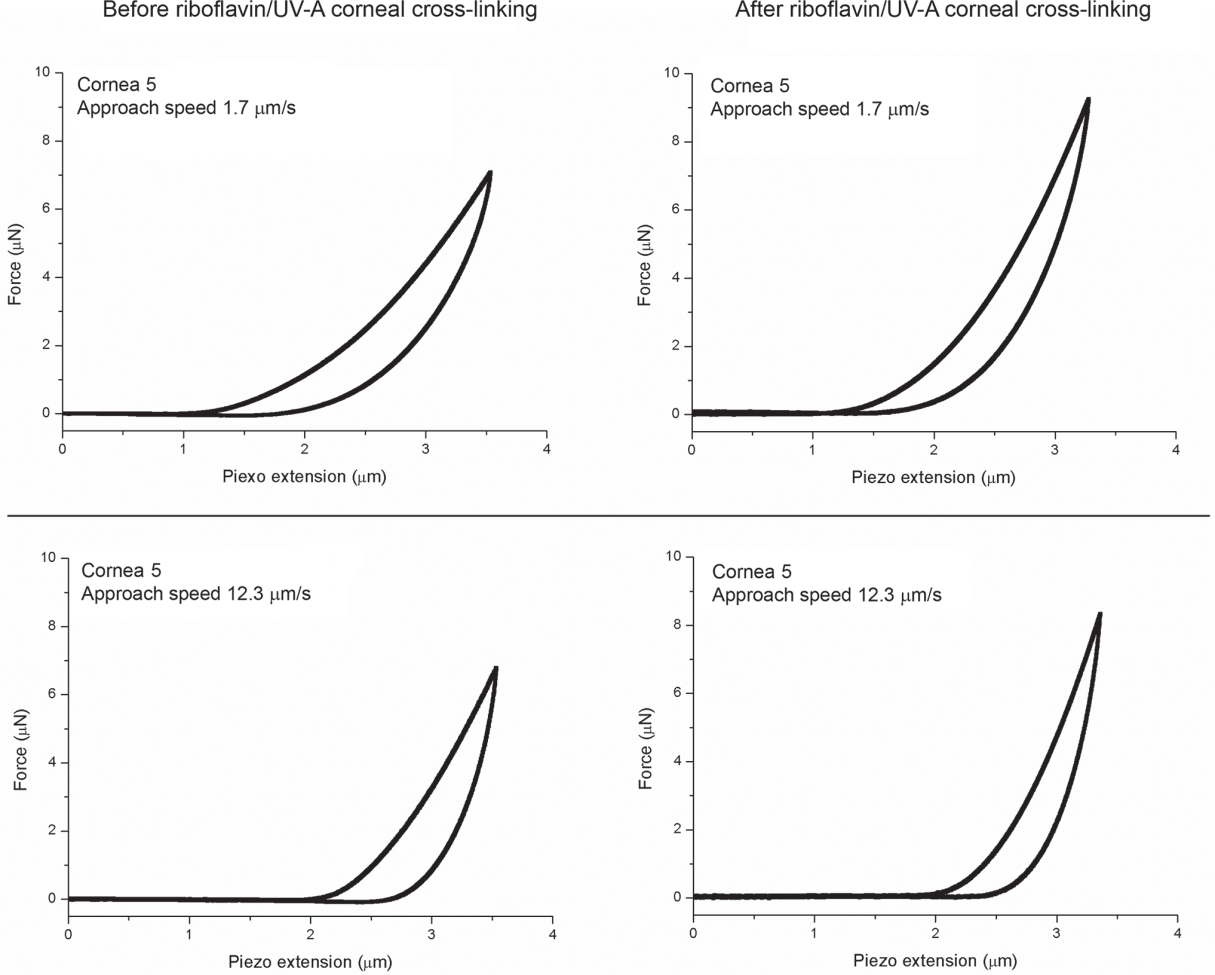
Representative force-indentation curves obtained before and after riboflavin/UV-A corneal cross-linking. In cornea 5, the force curves were acquired at approach speeds of 1.7- and 12.3-μm/s both before (left column) and after (right column) riboflavin/UV-A corneal cross-linking. After treatment, the approach curves became steeper, thus demonstrating an increased stiffness of the stroma; in addition, hysteresis decreased at the scale of stromal molecular interactions.

In all native corneal lenticules, the Young’s modulus increased with increasing application rates, as summarized in Table 2. After riboflavin/UV-A corneal cross-linking, the Young’s modulus increased by a mean of 1.1–1.5 times (Fig 2), except for sample n. 4 at an approach speed of 12.3 μm/s (Table 2). With this exception, the increase of *E* was statistically significant (P<0.05) at all application rates in all tissues. Representative force curves obtained before and after riboflavin/UV-A corneal cross-linking are shown in Fig 3. After chemical cross-linking with glutaraldehyde, the *E* values increased by a mean of 1.5–2.6 times (Table 2). The increase was greater than 2 times with respect to baseline values at the lower approach speeds (<8.2 μm/s) in both samples.

**Table 2:** Young’s modulus (*E*; MPa; M±SD) obtained for each sample before and after cross-linking.

	***E* values before riboflavin/UV-A cross-linking**					***E* values before glutaraldehyde cross-linking**	
**Approah speed** (μm/s)	**Cornea 1**	**Cornea 2**	**Cornea 3**	**Cornea 4**	**Cornea 5**	**Cornea 6**	**Cornea 7**
**1.7**	1.48±0.17	2.80±0.22	1.14±0.20	2.87±0.12	4.77±0.79	1.28±0.13	5.38±0.21
**3.5**	1.68±0.20	3.01±0.23	1.15±0.36	3.41±0.16	6.09±1.04	2.00±0.14	6.59±0.06
**8.2**	2.02±0.20	3.48±0.32	1.48±0.48	4.49±0.32	7.94±1.00	3.29±0.02	9.38±0.62
**12.3**	2.22±0.29	3.78±0.36	2.27±0.47	5.34±0.55	10.19±1.06	4.10±0.07	10.88±1.25
	***E* values before riboflavin/UV-A cross-linking**					***E* values before glutaraldehyde cross-linking**	
**Approach speed** (μm/s)	**Cornea 1**	**Cornea 2**	**Cornea 3**	**Cornea 4**	**Cornea 5**	**Cornea 6**	**Cornea 7**
**1.7**	2.21±0.42	3.26±0.20	1.62±0.34	3.82±0.37	7.46±0.15	5.10±0.18	10.81±0.36
**3.5**	2.54±0.29	3.59±0.14	1.63±0.33	4.12±0.32	9.11±0.21	5.61±0.17	11.50±1.26
**8.2**	2.98±0.62	4.03±0.18	2.13±0.41	4.69±0.18	12.44±0.50	6.31±0.01	11.39±1.23
**12.3**	3.28±0.59	4.21±0.32	2.77±0.40	5.14±0.13^#^	16.09±1.19	7.00±0.01	12.20±0.85

*P<0.05 between the pre- and post-treatment states (except for ^#^)

### 2. Hysteresis

The changes of the viscoelastic properties of the anterior stroma induced by cross-linking were evaluated by comparing the measurements obtained at specific ranges of applied loads both before and after treatment in each specimen. This procedure permitted to compare the hysteresis values with respect to the applied load, rather than the indentation depth, that was expected to change after cross-linking due to tissue stiffening.

**Fig 4:**
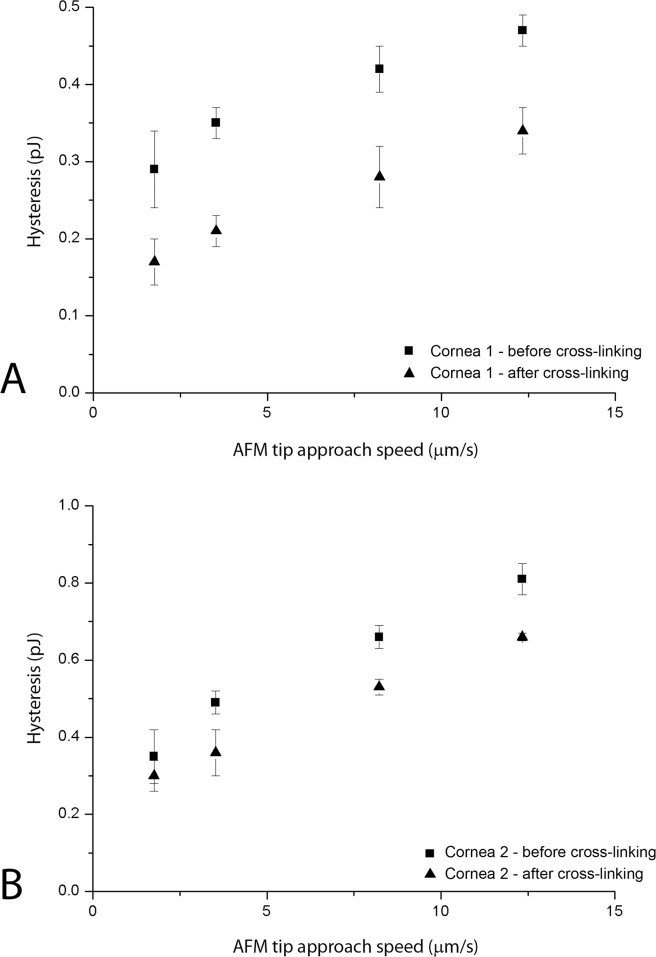
Hysteresis plotted as a function of the approach speed before and after riboflavin/UV-A corneal cross-linking. A) Hysteresis of cornea 1 at an applied load of 1.9 μN. B) Hysteresis of cornea 2 at an applied load of 3.6 μN. The changes of hysteresis of the anterior stroma were evaluated by comparing the measurements obtained at specific ranges of applied loads both before (squares) and after (triangles) treatment in each specimen. Bars indicate standard deviation.

**Fig 5:**
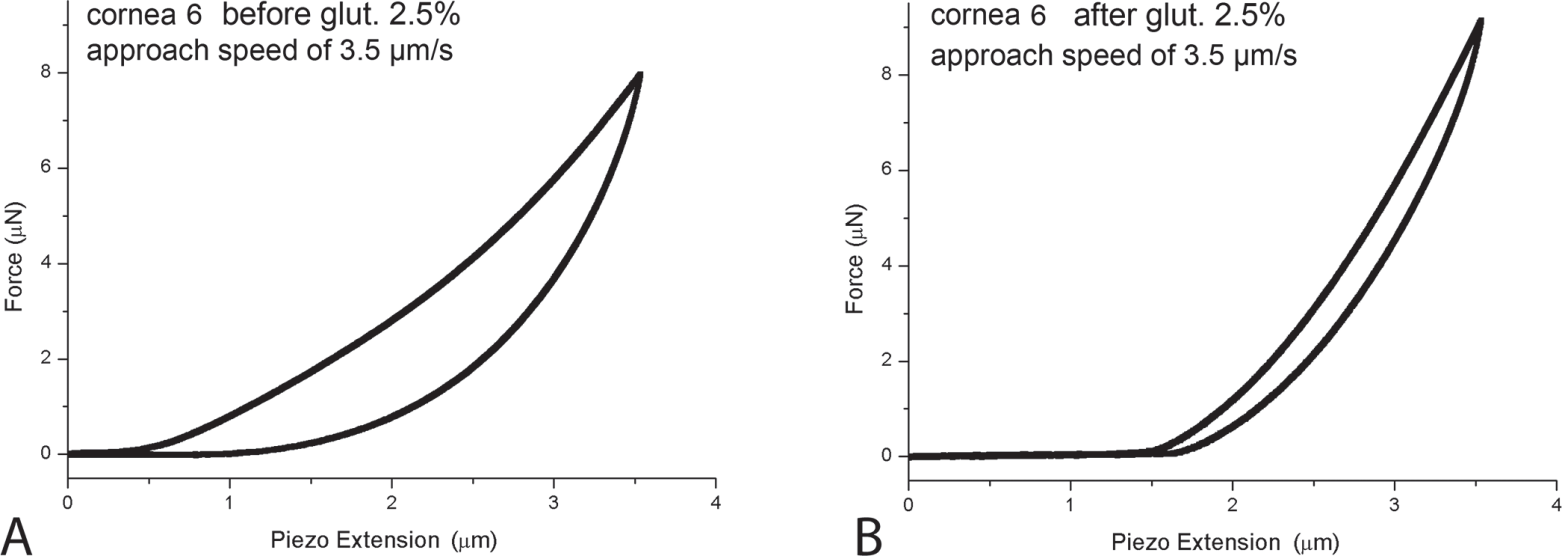
Representative force-displacement (F-D) curves before and after chemical corneal cross-linking using glutaraldehyde 2.5%. In cornea 6, the F-D curves were acquired before (A) and after (B) chemical cross-linking at an approach speed of 3.5 μm/s and an applied load of 6.5 μN. After treatment, the slope of the approach curve becomes steeper and the area under the curves becomes smaller than baseline measurements. The tip-stromal interaction demonstrates both an increase of the elastic response and a decrease of the viscous response of the chemically cross-linked stroma.

The hysteresis and the indentation depth values acquired in all specimens that underwent riboflavin/UV-A corneal cross-linking are summarized in Table 3. Both the hysteresis and the indentation depth decreased after treatment in all tissues; hysteresis decreased by a mean of 0.9 and 1.5 times with respect to baseline values (P<0.05). The highest decrease in hysteresis was found in cornea n. 1 (Fig 4A); the smallest decrease was found in cornea n. 2 (Fig 4B). After riboflavin/UV-A corneal cross-linking, the indentation depth decreased by a mean of 1.1–1.3 times. After cross-linking using glutaraldehyde, we found a dramatic decrease of *H*, ranging between 2.6 and 3.5 times with respect to baseline values (Fig 5). Accordingly, the indentation depth decreased by 1.2–1.6 times after glutaraldehyde cross-linking.

**Table 3:** Hysteresis and indentation depth values (M±SD) obtained at same ranges of applied loads before and after riboflavin/UV-A corneal cross-linking.

**Hysteresis** (pJ)										
	**Cornea 1** load range: 1.9±0.1 μN		**Cornea 2** load range: 3.6±0.1 μN		**Cornea 3** load range: 1.4±0.1 μN		**Cornea 4** load range: 4.5±0.1 μN		**Cornea 5** load range: 6.7±0.1 μN	
**Approach speed** (μm/s)	**Before treatment**	**After treatment** *	**Before treatment**	**After treatment** *	**Before treatment**	**After treatment** *	**Before treatment**	**After treatment** *	**Before treatment**	**After treatment** *
**1.7**	0.29±0.05	0.17±0.03	0.35±0.07	0.30±0.04	0.26±0.02	0.21±0.01	1.26±0.03	0.82±0.01	2.40±0.69	1.88±0.02
**3.5**	0.35±0.02	0.21±0.02	0.49±0.03	0.36±0.06	0.30±0.03	0.25±0.01	1.53±0.02	0.99±0.02	2.36±0.04	1.78±0.03
**8.2**	0.42±0.03	0.28±0.04	0.66±0.03	0.53±0.02			1.47±0.02	1.16±0.01		
**12.3**	0.47±0.02	0.34±0.03	0.81±0.04	0.66±0.01						
**Indentation depth** (μm)										
	**Cornea 1** load range: 1.9±0.1 μN		**Cornea 2** load range: 3.6±0.1 μN		**Cornea 3** load range: 1.4±0.1 μN		**Cornea 4** load range: 4.5±0.1 μN		**Cornea 5** load range: 6.7±0.1 μN	
**Approach speed** (μm/s)	**Before treatment**	**After treatment**	**Before treatment**	**After treatment**	**Before treatment**	**After treatment**	**Before treatment**	**After treatment**	**Before treatment**	**After treatment**
**1.7**	2.40±0.04	1.97±0.20	2.46±0.04	2.18±0.08	2,03±0.63	1.33±0.04	2.75±0.04	2.17±0.05	2.12±0.28	1.97±0.04
**3.5**	2.21±0.05	1.85±0.09	2.35±0.02	2.03±0.04	1.45±0.30	1.28±0.11	2.50±0.04	2.01±0.02	1.60±0.02	1.38±0.05
**8.2**	2.02±0.03	1.72±0.13	2.24±0.03	1.89±0.03			2.10±0.01	1.97±0.04		
**12.3**	1.93±0.02	1.69±0.20	2.15±0.01	1.86±0.03						

* Hysteresis values: P<0.05 between the pre- and post-treatment states

## Discussion

The efficacy of riboflavin/UV-A corneal cross-linking is primarily based on laboratory data demonstrating that the procedure increases the stiffness of the treated cornea. The effect of cross-linking on the mechanical properties of the cornea has been evaluated via different methods and techniques, which included strip extensiometry, inflation testing of corneal tissues or whole eye globes, scanning probe techniques etc. [12–17, 24–27]. Inflation and strip extensiometry techniques provide a convolved measure of the modulus of elasticity of the corneal tissue, while additional cross-link bonds between stromal proteins have been found to occur mainly across the anterior half stroma. Thus, a stiffening gradient effect is expected from the anterior to the posterior stroma after cross-linking [12,13,16,28]. To assess the effect of riboflavin/UV-A corneal cross-linking locally, the biomechanical response should be measured across different layers of the cornea. Atomic force microscopy can measure the biomechanical properties of the cornea at different stromal depths with high accuracy [14–20].

In the present study, a commercial AFM was used to probe the viscoelastic properties of the most anterior stroma of the human cornea. Each sample was compared independently from the others because of the inter-individual variation in biomechanical properties [18]. Conical AFM tip with 10 nm radius were used to indent individually the stromal collagen fibers (30 nm width). The methodology permitted to indent the anterior stroma up to 2.8 μm depth, thus investigating the mechanical behaviour of the most anterior collagen bundles under the Bowman’s layer. In addition, relatively high scan rates and loads were used in order to investigate the local viscoelastic response of the tissue.

Before treatment, the modulus of elasticity was rate dependent in all tissues, as previously discussed [18]. The larger elastic modulus found in our work with respect to previous AFM studies reflects mainly the modulus of the collagen fiber [18]. After riboflavin/UV-A cross-linking, the Young’s modulus of the anterior stroma increased significantly in all specimens, except for cornea n.4 at an approach speed of 12.3 μm/s. Apart from this exception, the *E* values increased by a mean of 1.1–1.5 times with respect to baseline values at all scanning rates. The lack of stiffening effect in one tissue at the highest approach speed was considered as a part of the inter-sample variability in the response to riboflavin/UV-A cross-linking. After chemical cross-linking using glutaraldehyde 2.5% solution, *E* increased by a mean of 1.5–2.6 times with respect to baseline values. This increase was higher at slow scanning rates, i.e., at the ranges where the nano-indentation response under loading was mainly determined by the elastic properties of the stroma [18].

In all native stromal samples, hysteresis increased with faster loading applications, as previously found [18,29,30]. This rate-dependent behaviour is typical of viscoelastic biological tissues. After riboflavin/UV-A cross-linking, stromal hysteresis decreased by a mean of 0.9–1.5 times with respect to baseline values in all samples. A great decrease in hysteresis, ranging between 2.6 and 3.5 times with respect to baseline values, was found after glutaraldehyde cross-linking. Since we evaluated hysteresis at different ranges of applied loads in each tissue, we were unable to compare these data between specimens. It is, however, well known that hysteresis decreases with age [30].

Recently, scanning microscopy techniques have been used to evaluate the changes of the elastic properties of porcine and human corneas after riboflavin/UV-A cross-linking [15–17]. Dias et al. [16] used micrometer-sized spherical indenters (59–74 μm diameter) and 10.4 N/m cantilever to probe the stroma at different depths. The authors used an approach speed of 15 μm/s with a maximum indentation depth of 6 μm, showing that the anterior stroma stiffened significantly after treatment, while the posterior stroma did not. Seifert et al. [17] used a 0.98 μm radius spherical tip and 4.01 N/m cantilever to indent sagittal stromal cryosections with a thickness of 16 μm. Overall, the results from previous AFM studies have provided quantitative information on the depth-dependent profiles of the Young’s modulus, showing that the stiffening effect of corneal cross-linking is mainly limited to the anterior 220 μm stromal depth. Beshtawi et al. [15] used scanning acoustic microscopy to explore the biomechanical changes induced by cross-linking in donor human corneas. The speed of sound in the anterior region between the cross-linked and control corneas was increased by a factor of 1.05, in fair accordance with this work. The increase of the speed of sound in the cross-linked corneas was depth-dependent, decreasing from the anterior stroma to the posterior part of the stroma. A sharp drop was noted in the region between 300 and 400 μm followed by a gradual decrease toward the posterior stroma.

Type I collagen is the most abundant structural component in corneal stroma. It is composed of three polypeptide chains and two types of single chains, α_1_ and α_2_, both of which are accessible to polymerization. Dimers of α chains are called β-components; trimers of α chains are called γ-components [31]. In this work, we compared the stiffening effect of riboflavin/UVA cross-linking to chemical cross-linking on human corneal stroma at a scale relevant to the size of single collagen fibers. Glutaraldehyde cross-linking of the cornea has been shown to increase the area associated with each corneal collagen molecule (intermolecular spacing) by 11% [29,32]. This observation can be a consequence of the type of polymerization induced by glutaraldehyde in collagen tissues. Glutaraldehyde reacts with the free amine groups of lysine or hydroxylysine amino acid residues of the polypeptide chains to form Schiff base intermediates from which several subsequent reactions may occur before a crosslink is formed [33,34]. Overall, the hydrolysable Schiff bases are stabilized by further reactions with other glutaraldehyde molecules during the formation of crosslinks, therefore glutaraldehyde introduces free aldehyde groups to the fixed tissue. On the contrary, riboflavin/UV-A cross-linking does not involve any addition of molecular residues; this is in fair accordance to what shown by Hayes et al. [9]. These authors, by using X-ray scattering, demonstrated that riboflavin/UV-A corneal cross-linking does not produce any significant change in the average fibril diameter or intermolecular spacing. However, the fibril spatial order factor was significantly higher in the cross-linked corneas when compared to untreated porcine corneas. The authors have thus postulated that the increase in short range order of collagen fibrils following riboflavin/UVA corneal cross-linking might occur as a result of cross-links being formed within the fibril coating, i.e., within and between proteoglycan core proteins. In accordance with this hypothesis, Zhang et al. [8] have demonstrated a preferential formation of covalent bonds between collagen, decorin and mimecan. Riboflavin/UV-A corneal cross-linking could involve cross-linking of collagen molecules with themselves, proteoglycan core proteins with themselves and collagen molecules with the two specific proteoglycan core proteins, mimecan and decorin. Thus, *in vivo* the formation of additional cross-linking bonds between collagen molecules and proteoglycan core proteins may contribute substantively to increase tissue strength. In our work, we aimed to understand the biomechanical effect of the procedure at the scale of stromal molecular interactions and demonstrated both an increase of the Young’s modulus and a decrease of hysteresis. These results are consistent with the formation of additional cross-linking bonds between stromal proteoglycan core proteins and collagen in riboflavin/UV-A corneal cross-linking. In keratoconus corneas, the altered collagen fibril arrangement of the anterior stroma can be positively regulated by the formation of additional bonds between the proteoglycan macromolecules and collagen, thus improving the long-lasting collagen-matrix resistance to intraocular pressure load [35].

Adequate control on tissue thickness before and during biomechanical experiments has been shown to provide predictive results on the corneal behaviour [14,18,36–38]. Tissue thinning is the main macroscopic tissue change after stromal soaking with dextran enriched riboflavin administration, even prior to UV-A irradiation, because of the high solution hyperosmolarity. Previous work [39] has shown a relative decrease in stromal hydration of bovine corneas of 5.5% and 8.5% after 20% dextran riboflavin only and cross-linking treatment respectively. With decreasing corneal hydration the Young’s modulus may increase [36]. This phenomenon can be explained based on the microstructure of the stroma. As the tissue thickness becomes smaller, the distance between negative charges become smaller and the net negative charge density inside the tissue increases. Therefore, decreasing the tissue thickness results in measuring a higher compressive force [40]. To avoid any bias due to changes in the hydration state during experiment, the corneal tissues were kept in 20% dextran solution before riboflavin soaking and after UV-A irradiation. Based on the studies of Hamaoui et al. [41] and our previous work [14,18,39], it was found that 20% dextran solutions were effective in avoiding tissue swelling and maintaining corneal hydration during experimentation of riboflavin/UV-A corneal cross-linking. In addition, only stromal soaking with 20% dextran enriched riboflavin solution has been shown to be ineffective to increase the stromal stiffness [13,42]. In this study, we indented less than 3 μm of the most anterior stroma (on average 6% of the whole stromal thickness); the change of hydration was therefore considered negligible.

In conclusion, we provided the first evidence that riboflavin/UV-A corneal cross-linking induces both an increase of the elastic response and a decrease of the viscous response of the most anterior stroma at the scale of stromal molecular interactions. These results are consistent with the knowledge that the formation of additional cross-linking bonds may occur at preferred sites of the surface of collagen fibrils and proteoglycan core proteins. From the present understanding, the term “corneal collagen cross-linking” (also known as CXL) is not fully descriptive of the basic mechanism of the procedure; it could be preferable to limit the term to a more comprehensive “corneal cross-linking” [43].
